# Be careful with triage in emergency departments: interobserver agreement on 1,578 patients in France

**DOI:** 10.1186/1471-227X-11-19

**Published:** 2011-10-31

**Authors:** Anne-Claire Durand, Stéphanie Gentile, Patrick Gerbeaux, Marc Alazia, Pierre Kiegel, Stephane Luigi, Eric Lindenmeyer, Philippe Olivier, Marie-Annick Hidoux, Roland Sambuc

**Affiliations:** 1 Laboratoire de Santé Publique, Faculté de Médecine, Equipe de recherche EA 3279 "Evaluation hospitalière-Mesure de la santé perçue", Marseille, France; 2 Service d'Accueil des Urgences, Hôpital de La Conception, Marseille, France; 3 Service d'Accueil des Urgences, Hôpital Sainte Marguerite, Marseille, France; 4 Service d'Accueil des Urgences, Hôpital du Pays d'Aix, Aix en Provence, France; 5 Service d'Accueil des Urgences, Centre Hospitalier Général, Martigues, France; 6 Service d'Accueil des Urgences, Hôpital Saint Joseph, Marseille, France; 7 Service d'Accueil des Urgences, Hôpital Henri Duffaut, Avignon, France; 8 Service d'Accueil des Urgences, Centre Hospitalier Général, Gap, France

## Abstract

**Background:**

For several decades, emergency departments (EDs) utilization has increased, inducing ED overcrowding in many countries. This phenomenon is related partly to an excessive number of nonurgent patients. To resolve ED overcrowding and to decrease nonurgent visits, the most common solution has been to triage the ED patients to identify potentially nonurgent patients, i.e. which could have been dealt with by general practitioner. The objective of this study was to measure agreement among ED health professionals on the urgency of an ED visit, and to determine if the level of agreement is consistent among different sub-groups based on following explicit criteria: age, medical status, type of referral to the ED, investigations performed in the ED, and the discharge from the ED.

**Methods:**

We conducted a multicentric cross-sectional study to compare agreement between nurses and physicians on categorization of ED visits into urgent or nonurgent. Subgroups stratified by criteria characterizing the ED visit were analyzed in relation to the outcome of the visit.

**Results:**

Of 1,928 ED patients, 350 were excluded because data were lacking. The overall nurse-physician agreement on categorization was moderate (kappa = 0.43). The levels of agreement within all subgroups were variable and low. The highest agreement concerned three subgroups of complaints: cranial injury (kappa = 0.61), gynaecological (kappa = 0.66) and toxicology complaints (kappa = 1.00). The lowest agreement concerned two subgroups: urinary-nephrology (kappa = 0.09) and hospitalization (kappa = 0.20). When categorization of ED visits into urgent or nonurgent cases was compared to hospitalization, ED physicians had higher sensitivity and specificity than nurses (respectively 94.9% versus 89.5%, and 43.1% versus 30.9%).

**Conclusions:**

The lack of physician-nurse agreement and the inability to predict hospitalization have important implications for patient safety. When urgency screening is used to determine treatment priority, disagreement might not matter because all patients in the ED are seen and treated. But using assessments as the basis for refusal of care to potential nonurgent patients raises legal, ethical, and safety issues. Managed care organizations should be cautious when applying such criteria to restrict access to EDs.

## Background

In the past 30 years, the number of visits to emergency departments (EDs) has increased, inducing overcrowding in many countries [1]. ED overcrowding is related to multiple complex problems: overburdened inpatient facilities, inadequate ED space, insufficient staffing, and inaccessibility to primary care services [2-6]. ED overcrowding has resulted in a longer stay in the ED and worse outcomes for persons who truly require emergency care [2,7,8].

Several review of the emergency medicine literature regarding EDs use and access to care over the past 30 years reveals significant evolution [9,10]. Indeed, concerns have been raised in several countries about the increasing numbers of patients attending EDs [1,11,12] with particular attention given to "inappropriate" or "nonurgent" ED use [13-15].

Using ED, rather than primary care settings, for nonurgent care contributes to the phenomenon of ED overcrowding [10]. This can reduce the continuity of care and impair preventive care and appropriate therapy for chronic conditions [14-17].

To resolve ED overcrowding and decrease the number of nonurgent ED patients, many solutions have been proposed [18], such as educational interventions recommending people should seek other sources of care before considering ED [19-23] or implantation of "gatekeepers" who require patients to have authorization from their primary care provider before going to the ED [24,25]. The most common solution has been for a nurse to triage the ED patients to identify potentially nonurgent patients, i.e. which could have been dealt with by general practitioner (GP) [20]. The main objective of triage is to assign a degree of urgency to patients depending on their complaint severity. In most of cases, the triage process is used to determine the priority of treatment in the ED. But many authors have proposed using the triage process to refer nonurgent patients to alternative sites of care [5,22,19,26].

Refusing care to nonurgent ED patients or referring them to alternative sites for care raises legal, ethical, and safety issues. Because there is no consensual method of triage, it is impossible to reliably and reproducibly identify nonurgent ED patients, as evidenced by the variability of proportions of such patients in the literature (from 4.8% to 90%) [10,19] and by the poor agreement between different methods of triage for the same patient group [10].

The objective of our study was to measure agreement on the urgency of an ED visit between the points of views of triage nurses and ED physicians. Second, we sought to determine if the level of agreement is consistent among different sub-groups based on following explicit criteria: age, medical status, and type of referral to the ED.

## Methods

### Study Design and Setting

A multicentric cross-sectional study was conducted over a 3-day period (a weekday and two weekend days), in April 2007, in a sample of EDs located in the Provence-Alpes-Côte d'Azur (PACA) region, in France. This region has a population of 4.8 million which represent 7.6% of the population of France, and covers 34,400 km^2 ^with population densities from 153 persons per km^2 ^[27]. The PACA region is served by a total of 53 EDs, which treated between 11,000 and 65,000 ED patients per year. The distribution of 53 EDs allows to 99% of the population access to an ED in less than 45 minutes (and 85% in less than 15 minutes). Private (17%) and public (83%) hospitals were represented.

The 53 EDs were classified according to the following two strata: the number of annual visits to these EDs (high attendance (25 000 or over visits per year) and medium or low attendance (less than 25 000 visits per year)) and, the geographical location of EDs (located urban area characterized by higher population density with at least 2 000 residents and by the urban-type land use, not allowing any gaps of typically more than 200 meters [28] or not). Finally, 17 EDs were randomly selected among the 53 EDs according the two strata. Table 1 describes the characteristics of these 17 EDs.

**Table 1:** Hospital Characteristics

Hospital	Type of hospital	Location	Annual ED visits (Means)	NU patients in the study sample (n)
H1	Tertiary	Urban	24,500	98
H2	Secondary	Urban	17,500	62
H3	Secondary	Rural	16,000	72
H4	Teaching/Tertiary	Urban	48,000	171
H5	Teaching/Tertiary	Urban	59,000	122
H6	Teaching/Tertiary	Urban	31,500	127
H7	Tertiary	Urban	33,500	31
H8	Tertiary	Urban	53,000	172
H9	Secondary	Urban	32,000	99
H10	Secondary	Rural	20,500	100
H11	Tertiary	Urban	36,500	113
H12	Tertiary	Urban	34,000	22
H13	Secondary	Urban	33,000	47
H14	Secondary	Rural	29,000	83
H15	Secondary	Rural	18,000	47
H16	Secondary	Rural	12,500	44
H17	Tertiary	Urban	44,000	168

H = hospital; NU = nonurgent

### Population and Data collection

All patients aged 18 years and older who presented in one of participant EDs between the hours of 8 AM and 12 midnights were included. Study hours were limited because of few patients come after midnight [29]. Patients were excluded if they required immediate medical care and had communication difficulties.

Immediately after the admittance and nurse triage, all patients agreeing to participate were interviewed face to face in the ED by a trained research assistant who was not involved in care. The anonymous standardized questionnaire collected the following variables [Additional file 1]:

- Patients' characteristics: demographic (age, sex) and socio-economic characteristics (employment status, health insurance status), utilization of health care services (having a primary care physician, Yes/No response from the patient), health status (suffering from chronic disease, Yes/No response from the patient).

- ED visit characteristics: type of referral to the ED (self-referral, health care professional or other referral i.e. police, ambulance, employer, school, sports facility), chief complaint, duration of the presenting complaint, and mode of arrival. At the end of the ED consultation, the research assistant collected if the patient had diagnostic tests and treatments performed in the ED, and visit disposition (hospitalization).

Moreover, during their activity, trained triage nurses, after the admittance, and trained ED physicians, immediately at the end of the consultation, were asked to complete the questionnaire for each patient seen [Additional file 2]. They independently gave their expert opinion concerning the urgency of the admission of the patient. All ED health professionals had at least one year' experience of the ED.

#### Categorization of the urgency of the ED admission

According to the literature review, patients categorized as nonurgent are defined as those "who could have been dealt with by general practitioner" [10].

The categorization was conducted in two times and from two categories of ED health care professionals. Triage nurses, immediately after the admittance, and ED physicians, immediately at the end of the consultation, were asked to answer the rhetorical question, "Could this problem be taken care of by a primary care physician?" and, if the answer was yes, the ED visit was categorized as "nonurgent".

First, nurses conducted their triage interviews in the usual manner, i.e. without the use of written protocols or algorithms. The categorization was only done from a brief interview of the patient and included patient complaint(s). Second, as triage nurses, the ED physicians' categorization was done without the use of written protocols or algorithms. But the categorization was done from clinical examination, medical record, results of diagnostic tests, and from treatment performed in the ED. This categorization was performed in blind; ED physician raters did not have access to the triage nurses' notes.

For each patient, categorization was performed in usual conditions without disturbing the activity of ED health professionals. Triage nurses had not attended training session specifically for this study; however categorization of urgency is part of their qualifications [30].

### Data analysis

Data were analyzed on Spss 16.0 by using proportions or means, and standard deviations of all variables. The main outcome variable was whether the ED visit was urgent or not.

To evaluate the level of agreement on triage categories between nurses and ED physicians, we calculated the chance-adjusted measure of agreement (Kappa-value) from 4 × 4 tables. Qualitative descriptions of agreement were as follows: 0.81-1.0 = "almost perfect", 0.61-0.80 = "substantial", 0.41-0.60 = "moderate", 0.21-0.40 = "fair", 0.0-0.20 = "slight" [31]. Kappa-values are reported with 95% confidence intervals (CIs). Sensitivity, specificity, and positive and negative predictive values (PPV and NPV) of accuracy of categorization into urgent and nonurgent case between triage nurses and ED physicians who were the reference. To assess the discrimination power of this model, a receiver operating characteristic (ROC) curve was constructed. The ROC curve is a graphic method for indicating the trade-off between the true-positive rate (sensitivity) and the false-positive rate (1 - the specificity) of a test or diagnostic manoeuvre. Generally, the most discriminating tests have the largest area under the ROC curve, the maximum being 1.0 [32]. Moreover, sensitivity, specificity, PPV and NPV of decision for hospitalization were calculated. For these analyses, ED patients hospitalized at the end of the consultation were compared with patients categorized into urgent or nonurgent cases by triage nurses and by ED physicians.

### Subgroup analysis

Analyses of agreement were performed within subgroups stratified by explicit criteria. Subgroups were defined according to the following criteria:

- Age: 75 years or older versus ager younger than 75 years,

- Chief complaints recorded in 22 subgroups of case mix based on the "French Emergency Nurses Classification" [33],

- Suffering from chronic disease versus none,

- Duration of presenting complaints: 24 hours or less versus more than 24 hours,

- Mode of arrival: own transport versus ambulance transport,

- Type of referral to the ED recorded in 3 subgroups: self-referral, health care professional, other referral.

Kappa-values with 95% CI were analyzed within all these subgroups.

### Ethical Considerations

Our study is a non interventional research and does not need to be approved by an ethics committee under the criteria of the bioethics law. So, our study does not require the authorization of the National Commission for Informatics and Freedom due respect for patient anonymity [34].

## Results

During the study period, 1,949 adult patients visiting the 17 emergency departments were eligible for the study and 1,928 were included (98.9%). EDs received a mean of 113.4 adult patients ± 48.1 (median = 103, minimum = 31 and maximum = 172).

Of the 1,928 patients included, 350 were excluded from the analysis because data were not available from both triage nurses and ED physicians. The final study sample comprised 1,578 patients for whom two assessments were obtained.

### Demographic characteristics and insurance status of ED patients [Table 2]

**Table 2:** Characteristics of the study population

Characteristics	n	%
**Sex**	**1,577 **	**99.9**
Male	827	52.4
Female	750	47.6
**Age (years)**	**1,575 **	**99.8**
18-24	344	21.8
25-44	525	33.3
45-64	357	22.7
65-74	124	7.9
≥ 75	225	14.3
**Employment status**	**1,432 **	**90.7**
Employed	754	52.7
Retired	475	33.2
Unemployed	203	14.2
**Health insurance status**	**1,515 **	**96.0**
Uninsured	13	0.9
Primary health insurance with supplementary coverage	1,303	86.0
Primary health insurance without supplementary coverage	199	13.1
CMUC* among patients having supplementary coverage (n = 1,303)	**1,286**	**98.7**
Yes	134	10.4
No	1,152	89.6
**Having a primary care physician**	**1,573**	**99.7**
Yes	1,461	92.9
No	112	7.1
**Suffering from chronic disease**	**1,572**	**99.6**
Yes	577	36.7
No	995	63.3

* CMUC: French health insurance specifically for individuals and families with low incomes and resources

Of the 1,578 patients included in the study, 52.4% were males and the mean age of ED patients (± standard deviation (SD)) was 45.2 years ± 21.4 (from 18 to 100 years); 14.3% of patients were 75 years old and over. Most patients had primary health insurance with supplementary coverage (86.0%); 10.4% of them were covered by French health insurance specifically for individuals and families with low incomes and resources (named "CMUC"). The majority of included patients were followed by a general practitioner (92.9%). More than one third suffered from chronic disease (36.7%).

### Characteristics of ED visits [Table 3]

**Table 3:** Characteristics of the ED visits

Characteristics	n	%
Chief complaint*	**1,577**	**99.9**
Traumatic	763	48.4
Non traumatic	814	51.6
**Duration of the presenting complaint**	**1,573**	**99.7**
≤ 24 hours	1,223	77.7
> 24 hours	350	22.3
**Mode of arrival **	**1,572**	**99.6**
Own transport	1,013	64.4
Ambulance transport	559	35.6
**Referral to the ED**	**1,571**	**99.6**
Self-referral	996	63.4
Primary care physician/Other health professional	270	17.2
Other referral (police, ambulance, employer, school, sports facility)	305	19.4
**Diagnostic tests performed in the ED**	**1,570**	**99.5**
Yes	1,074	68.4
No	496	31.6
**Treatment performed in the ED**	**1,564**	**99.1**
Yes	926	59.2
No	638	40.8
**ED visit disposition**	**1,553**	**98.4**
Hospital admission	352	22.7
Home	1,201	77.3

*Details of chief complaints are presented in Table 4.

Presenting complaints had lasted less than 24 hours for 77.7% of patients. Only 17% had been referred to the ED by a primary care physician. The others were self-referred (63.4%) or referred for medico-legal reasons (19.4%) (Employer, school, police...).

More than half of patients were consulting the ED for non-trauma complaints. Nearly two thirds of ED patients received diagnostic tests; 59.2% received treatment in the ED, and 22.7% were hospitalized.

### Variability in the proportions of nonurgent ED visits and overall agreement between triage nurses and ED physicians

**Figure 1:**
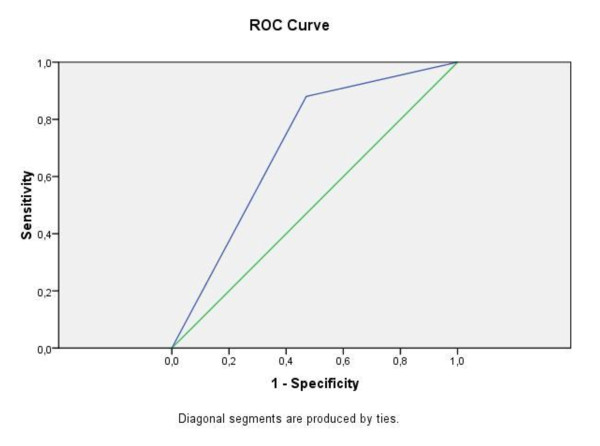
**Receiver operating curve (ROC) and area under curve (AUC) values for categorization into urgent and nonurgent case between triage nurses and ED physicians who were the reference**.

Of the 1,578 ED visits, the proportion of nonurgent ED patients was 26% according to triage nurses upon the entry, and 34.3% according to ED physicians at the end of the consultation (p < 0.001, Table 4). Overall level of agreement was moderate (kappa = 0.43 ± 0.02; 95% CI, 0.39% to 0.48%). The model showed a high sensitivity of 88.0% (Table 5). The area under the ROC curve was 0.70 with 95% CI 0.68 to 0.73 (Figure 1).

**Table 4:** Triage agreement between nurses and ED physicians

		Categorization conducted by physicians, at the end of the ED consultation*		
		Urgent, n (%)	Nonurgent, n (%)	Total, n
**Categorization conducted by triage nurses, after the ED admittance**	Urgent	912 (88.0)	255 (47.0)	1,167
	Nonurgent	124 (12.0)	287 (53.0)	411
	Total, n	1,036	542	1,578

*Column percentages

p < 0.001

Kappa = 0.43; 95% CI = 0.39-0.48

**Table 5:** Sensitivity, specificity, and predictive value in prediction of urgent or nonurgent cases

	Sensitivity (%)	Specificity (%)	Positive Predictive Value (%)	Negative Predictive Value (%)	Number of patients
**Triage nurse versus ED Physician**	88.0	52.9	78.1	69.8	1,578

Table 4 shows the distribution between triage categories determined by triage nurses upon the entry to the ED and by ED physicians at the end of the consultation. Of the 1,036 patients categorized as urgent by ED physicians, 124 (12%) of them were categorized as nonurgent by triage nurses. These 124 patients were, for the majority, women (54%), self-referred (68.0%) and suffering from a medical problem for more than 24 hours (29.0%).

### Variability in agreement between triage nurses and ED physicians within subgroups from explicit criteria characterizing the ED visit

Within the 17 EDs, the levels of agreement were variable, ranging from 0.21 to 0.71. The highest kappa value concerned an ED with the smallest number of patients (n = 31).

Table 6 shows results of analyses in subgroups. The levels of agreement within all subgroups based on explicit criteria were low (from moderate to slight) except in 3 subgroups of case mix.

**Table 6:** Subanalyses of agreement of explicit criteria

	**Total cases **n (%)	**Disagree **(n)		**Agree **(n)	Kappa	95% CI
		**Physicians (Urgent) - Nurses (Nonurgent)**	**Nurses (Urgent) - Physicians (Nonurgent)**			

**Age**						
< 75 years	1,350 (85.7)	102	232	1,016	0.44	0.39-0.49
≥ 75 years	225 (14.3)	22	22	181	0.29	0.13-0.45
**Non-traumatic complaints**	**814 (51.6)**	**66**	**130**	**618**	**0.46**	0.40-0.52
Cardiovascular	157 (9.9)	9	23	125	0.25	0.06-0.43
Gastrointestinal	131 (8.3)	14	22	95	0.35	0.18-0.52
Rheumatology	114 (7.2)	12	23	79	0.36	0.19-0.53
ENT	59 (3.7)	2	12	45	0.52	0.32-0.73
Urinary nephrology	55 (3.5)	7	6	42	0.09	-0.20-0.39
Neurology	39 (2.5)	3	7	29	0.34	0.02-0.66
Respiratory	41 (2.6)	5	2	34	0.48	0.16-0.81
Ophthalmology	43 (2.7)	5	6	32	0.26	-0.07-0.59
Infectious	32 (2.0)	1	4	27	0.60	0.30-0.90
Endocrine	31 (2.0)	4	7	20	0.22	-0.13-0.56
Psychiatric	25 (1.6)	-	6	19	0.53	0.24-0.82
Toxicology	19 (1.2)	-	-	19	1.00	-
Dermatology/Allergy	24 (1.5)	2	6	16	0.33	-0.02-0.69
Gynecological	20 (1.3)	1	2	17	0.66	0.31-1.00
Others (transfer, medical prescription renewal, technical problem probe)	24 (1.5)	1	4	19	0.58	0.27-0.89
**Traumatic complaints**	**763 (48.4)**	**57**	**125**	**581**	**0.40**	**0.33-0.47**
Limb/Pelvis injury	407 (25.8)	32	72	303	0.39	0.29-0.48
Wound	148 (9.4)	12	20	116	0.26	0.07-0.45
Facial/Neck/Thorax/Abdomen/Spinal Injury	80 (5.1)	7	12	61	0.49	0.29-0.69
Aggression	34 (2.2)	2	9	23	0.35	0.06-0.64
Cranial injury	21 (1.3)	1	1	19	0.61	0.12-1.10
Others (burn, subcutaneous foreign body)	73 (4.6)	3	11	63	0.47	0.24-0.70
**Suffering from chronic disease**						
Yes	577 (36.7)	34	81	462	0.47	0.38-0.54
No	995 (63.3)	90	173	732	0.41	0.35-0.47
**Duration of the presenting complaint**						
≤ 24 hours	1,223 (77.7)	88	198	937	0.39	0.33-0.45
> 24 hours	350 (22.3)	36	56	258	0.47	0.38-0.56
**Mode of arrival**						
Own transport	1,013 (64.4)	87	178	748	0.45	0.40-0.50
Ambulance transport	559 (35.6)	37	77	445	0.23	0.13-0.33
**Referral to the ED**						
Self-referral	996 (63.4)	83	170	743	0.46	0.41-0.51
Health care professional	270 (17.2)	21	35	214	0.26	0.11-0.40
Other	305 (19.4)	18	49	238	0.30	0.18-0.43
**Outcome of the visit to the ED**						
Hospitalization	352 (22.7)	30	11	311	0.20	0.04-0.35
Home	1,201 (77.3)	93	239	869	0.42	0.37-0.47

The levels of agreement within the 22 subgroups of complaints were variable, ranging from 0.09 to 1.00. Among the 22 subgroups, 10 showed fair inter-observer agreement (k = 0.21-0.40) and 7 moderate agreement (k = 0.41-0.60). The lowest level of agreement concerned the subgroup of urinary-nephrology (k = 0.09, slight). The highest kappa-values concerned three subgroups of complaints: cranial injury (k = 0.61, substantial), gynecological complaints (k = 0.66, substantial) and toxicology complaints (k = 1.00, almost perfect).

For the other subgroups, levels of agreement were also low (from 0.20 to 0.47) and showed considerable variability. The lowest level of agreement concerned the subgroup of hospitalization (k = 0.20, slight) and the highest concerned the three following subgroups: duration of the presenting complaint (> 24 hours, k = 0.47), suffering from chronic disease (k = 0.47) and self-referral (k = 0.46). These three levels of agreement were moderate.

### Is that hospital admission is a relevant indicator to categorize patients into urgent or nonurgent cases?

Hospital admission is not a relevant indicator. The distribution of categorization of urgency relative to hospitalization status is shown in Table 7. Whatever the professional who conducted the categorization (triage nurse or ED physician), most urgent patients were not hospitalized. Among the 409 nonurgent patients identified by triage nurses, 9% were hospitalized. These patients had no specific characteristics. Similarly, among the 536 nonurgent patients identified by ED physicians, 18 were hospitalized (3.4%). The majority of these 18 patients were older (70%, mean age 69.2 years ± 4.7; median 79.5 years), and reported neuropsychological problems (20%) and alteration of clinical status (20%).

**Table 7:** Relationship between categorization of ED visits and hospitalization (n = 1,553)

	Hospitalization		
	**Yes** **n (%)**	**No** **n (%)**	**Total cases** n (%)*

**Triage nurse**			
Urgent patients	315 (27.5)	829 (72.5)	1,144 (73.7)
Nonurgent patients	37 (9.0)	372 (91.0)	409 (26.3)
**ED Physician**			
Urgent patients	334 (32.8)	683 (67.2)	1,017 (65.5)
Nonurgent patients	18 (3.4)	518 (96.6)	536 (34.5)
Total cases*	352 (22.7)	1,201 (77.3)	

* Percentages on line

When categorization of ED visits into urgent or nourgent cases was compared to hospitalization, ED physicians had higher sensitivity and specificity than nurses (respectively 94.9% versus 89.5%, and 43.1% versus 30.9%). Overall, for ED physicians and triage nurses, positive predictive values were low (32.8% versus 27.5%) and negative predictive values were higher (96.6% versus 90.9%) [Table 8].

**Table 8:** Sensitivity, specificity, and predictive value in prediction of hospitalization

	Sensitivity (%)	Specificity (%)	Positive Predictive Value (%)	Negative Predictive Value (%)	Number of patients
Triage nurse	89.5	30.9	27.5	90.9	1,553
ED Physician	94.9	43.1	32.8	96.6	1,553

## Discussion

Our study shows a moderate level of agreement between triage nurses and ED physicians in decisions to categorize patients in urgent or nonurgent cases. This finding corroborates the results of the previous studies of Brillman *et al*., Caterino *et al*., Frey *et al*., O'Brien *et al*., and Lowe *et al*., who used the same method and also found poor kappa levels of agreement [35-39]. Kelly *et al*. are the only ones who found a high level of agreement between nurses and ED physicians (kappa = 0.74), probably because the categorization performed by the nurses and physicians was conducted at the same time (after patients' discharge from the ED) and was based on chart review [40]. In our study, like in the others, categorization was performed at two times: upon the entry to the ED by triage nurses, and at the end of visit by ED physicians. Moreover, our data was collected from a representative sample, indeed the socio-demographic and ED visit characteristics were similar to those reported in the literature [6,10,29].

Whatever the subgroups stratified by explicit criteria, the level of agreement remained moderate, except for three subgroups of complaint: toxicology, gynecological and cranial injury subgroups. The high levels of agreement for these three subgroups can be explained by the homogeneneity of case mix. For example, the subgroup of toxicology concerned only two kinds of diagnoses: carbon monoxide poisoning and alcoholism.

We also found a low level of agreement for the sub-group of patients older than 75 years. Relative to younger ED patients, elderly patients have a complex mix of medical and social needs which increases the difficulty to categorize patients into urgent or nonurgent cases.

Our study shows a slight level of agreement between triage nurse and ED physicians within the subgroup of hospitalization. This finding corroborates previous studies [34,41] which have shown limitations in using the criterion of hospitalization as an outcome variable to categorize patients into nonurgent cases [2,34,41]. However, this variable is often chosen by authors because it is the only concrete outcome variable recognized as the surrogate indicator of the need for prompt care. The low predictive positive value found in our study corroborates that hospitalization is not a consistent outcome variable to categorize patients into urgent or nonurgent cases.

It is not defined that all urgent patients need hospitalization after ED consultation and/or that all nonurgent patients should be discharged to home. However, urgents patients with potentially serious complaints (chest or abdominal pain, asthma...) or serious clinical signs (hypoglycemia, persistent fever, alteration in blood pressure) may be investigated, treated, and discharged from the ED. Moreover, the decision to hospitalize a patient categorized as nonurgent may be somewhat subjective and at times based largely on multiple social, economic factors or because of deficiencies in downstream interventions that are specific to a particular patient population. In this case, hospitalized patients categorized as nonurgent by ED physicians could be described as inappropriate. The results showed that physicians were not influenced by the final disposition of hospitalization. Indeed, hospitalized patients categorized as nonurgent (n = 18) were elderly and cognitively impaired.

The finding of low agreement between triage nurses and ED physicians is due partly to the two times of categorization. Indeed, the categorization conducted by ED physicians at the end of the consultation have the benefit of information based on supplementary explicit criteria, like the results of diagnostic tests performed during the ED visit and/or a consultation with a specialist physician. Our objective is not to reconsider the role of the triage nurse; we recognize that a brief triage performed by a nurse cannot always predict whether the patient has an urgent problem or not. However, this finding highlights the potential unsafe of triage, especially if the objective of the triage is to redirect nonurgent patients outside the ED. Indeed, the risk is to inadvertently refuse care to patients who truly in need of emergency interventions.

### Limitations

Several potential limitations should be addressed. Firstly, while we examined in great detail the different sub-groups based on following explicit criteria: age, medical status, and type of referral to the ED, we did not analyze the impact of the trained ED health professionals themselves. We conducted the study with ED health care professionals present during the inclusion period, in the usual manner, i.e. without the use of written protocols or algorithms. However, in previous studies measuring level of agreement, training, experience, knowledge, and skill of ED health professionals did not influence kappa values [35,42,43]. The authors found substantial disagreement even among health care professionals with the same training. Secondly, when designing our test study, sample size calculation should have been performed in order to guarantee the design accuracy. But, we performed a sample size calculation retrospectively based on the methodology of Flack VF et al [44]. Data were analysed on PASS 2008. In a test for agreement between two raters using Kappa statistic, a sample size of 1,986 subjects achieves 80% power to detect a true Kappa value of 0.43 in test of null hypothesis: Kappa = 0.50 versus alternative hypothesis: Kappa <> 0.50 when there are two categories with frequencies equal to 0.70 and 0.30. This power calculation is based on a significance level of 0.050. Thus, we included 1,578 patients in our study. Moreover, we found six similar studies which compared different methods of categorization in the same population [35,40]. These articles showed considerable variability in levels of agreement between the different methods to categorize ED visits into nonurgent or urgent cases, ranging in κ value from 0.20 to 0.74. These studies did not perform a sample size calculation.

## Conclusions

This multicentric study of 1,578 adults on triage to identify nonurgent patients demonstrates triage conducted by nurses is not consistent. The lack of physician-nurse agreement and the inability to predict hospitalization have important implications for patient safety. When categorization of urgency is used to determine the priority of treatment into the ED, disagreement might not matter because all patients in the ED are seen and treated. When urgency assessments are used as the basis for refusal of care to potential ED patients, the uncertainly is a matter of greater concern. Therefore, considerable caution should be used when managed care organizations apply such criteria to restrict access to EDs.

## Competing interests

The authors declare that they have no competing interests.

## Authors' contributions

ACD and SG participated in the design and the coordination of the study, performed the statistical analysis and helped to draft the manuscript. SG, ACD, PG, MA, PK, SL, PO and MAH participated in the design of the study, interpreted the results and helped to draft the manuscript. RS participated in the statistical analysis and helped to draft the results. All authors read and approved the final manuscript.

## Pre-publication history

The pre-publication history for this paper can be accessed here:

http://www.biomedcentral.com/1471-227X/11/19/prepub

## Supplementary Material

Additional file 1 **Patient questionnaire**. Questionnaire used to assess the urgency of an ED visit and to explore factors associated or not with this assessment. Click here for file

Additional file 2 **ED physician questionnaire**. Questionnaire used to assess the ED visit. Click here for file
