# Supplementary material for: Be careful with triage in emergency departments: interobserver agreement on 1,578 patients in France
Source: BMC Emerg Med. 2011 Oct 31;11:19. doi: 10.1186/1471-227X-11-19 (PMC3215166; doi:10.1186/1471-227X-11-19)
Supplement: Additional file 1 — Patient questionnaire. Questionnaire used to assess the urgency of an ED visit and to explore factors associated or not with this assessment. [file 1471-227X-11-19-S1.DOC]

**Additional file 1**

Title: Patient questionnaire

Description: Questionnaire used to assess the urgency of an ED visit and to explore factors associated or not with this assessment.

#### PATIENT QUESTIONNAIRE

# Emergency Department : _____________________________

1. Date of visit : _____/____/______
2. Day of the week: ………………………
3. Time of arrival: ______ : _______

**CATEGORIZATION CONDUCTED BY THE TRIAGE NURSE**

**Immediately after the admittance**

1. Presenting complaint: ____________________________________________________________
2. Could this problem be taken care of by a primary care physician? ❑ Yes ❑ No
3. Why? _________________________________________________________________________

**CHARACTERISTICS OF THE ED VISIT**

1. Patient’s complaint(s), symptom(s), or other reason(s) for this ED visit (Use patient’s own words) ________________________________________________________________________________________________________________________________________________________________
2. Mode of arrival:

❑ Ambulance ❑ Public service (police, social service) ❑ Own transport ❑ Other: ________________

1. Who made the decision to come to the ED?

- My general practitioner
- Myself
- A member of my family
- My employer
- Other _______________

1. How much time has passed since symptoms began and the decision to come to the ED?

- One day
- Less than a week
- More than a week (note the period:__________)

1. Did you try to speak to a primary care provider before coming to the ED? ❑ Yes ❑ No
2. What made you choose to come to the ED today? (multiple answers possible with ranking 1 to X):

- My health problem require immediate attention
- My health problem is too urgent to wait to see a primary care provider
- My problem was too serious or to complex to see a primary care provider
- My problem require x-rays, laboratory testing or treatment
- I did not want my primary care provider to know about my health problem
- I’m afraid
- I'm in a hurry
- My primary care provider was unavailable.
- I need a medical certificate (administrative reasons)
- It is easier for me to come to the ED
- Not applicable (The patient does not choose to come)
- Other _________________ (1 word).

1. Do you suffer from any chronic disease or condition? ❑ Yes ❑ No
2. On an urgency scale from 1 to 20, how would you rate your current urgency?___/20

utilization of health care services

1. Currently, did you have a primary care provider? ❑ Yes ❑ No
2. What do you do when you have health problems?

- I usually see my primary care provider
- I see other physician
- I usually prefer self-medication without seeing the primary care
- I prefer to come to the hospital
- Other ____________________

1. In the past year, how many times have you consulted your primary care provider?

❑ None ❑ 1 or 2 times ❑ 3-5 times ❑ more than 5 times

1. In the past year, how many times have you been treated at an ED?

❑ None ❑ 1 or 2 times ❑ 3-5 times ❑ more than 5 times

PATIENT INFORMATION

1. Patient’s age : _________
2. Sex : ❑ Male ❑ Female
3. Employment status: ❑ Employed ❑ Unemployed

| 1. Currently, what is your primary medical insurance? | 1. What is your supplementary health insurance? |
| --- | --- |
| - None | - None |
| - « Sécurité sociale » *(French health insurance)* | - Private supplementary health insurance |
| - CMU *(French health insurance designed specifically to individuals and families with low incomes and resources)* | - Supplementary CMU *(French supplementary health insurance designed specifically to individuals and families with low incomes and resources)* |
