# Supplementary material for: Be careful with triage in emergency departments: interobserver agreement on 1,578 patients in France
Source: BMC Emerg Med. 2011 Oct 31;11:19. doi: 10.1186/1471-227X-11-19 (PMC3215166; doi:10.1186/1471-227X-11-19)
Supplement: Additional file 2 — ED physician questionnaire. Questionnaire used to assess the ED visit. [file 1471-227X-11-19-S2.DOC]

**Additional file 2**

Title: ED physician questionnaire

Description: Questionnaire used to assess the ED visit

**ED PHYSICIAN QUESTIONNAIRE**

**CATEGORIZATION CONDUCTED AT THE END OF THE CONSULTATION**

1. Date of ED visit : _____/____/______
2. Emergency department: _________________________
3. Physician’s diagnosis for this visit: _______________________________________________
4. Diagnostic tests performed in the ED (Imaging, blood tests, cultures, tests): ❑ Yes ❑ No
5. Treatment performed in the ED (Medications, injections): ❑ Yes ❑ No
6. Visit disposition: ❑ Hospitalization ❑ Home ❑ Other _______________
7. Could this problem be taken care of by a primary care physician? ❑ Yes ❑ No
8. Why? ________________________________________________________________________
